# Expert voices in the news reporting of the coronavirus pandemic: A study of UK television news bulletins and their audiences

**DOI:** 10.1177/14648849221127629

**Published:** 2022-12

**Authors:** Marina Morani, Stephen Cushion, Maria Kyriakidou, Nikki Soo

**Affiliations:** School of Journalism, 2112 Media and Culture - Cardiff University, Cardiff, UK

**Keywords:** television news, sources, experts, content analysis, news diary study

## Abstract

The study examines the role of experts in UK television news at the start of the coronavirus pandemic by analysing both how they were used in coverage and perceived by news audiences. Our systematic content analysis of sources (*N* = 2300) used in the UK’s flagship evening news bulletins found a reliance on political sources, principally from the government’s perspective. We also discovered health and scientific experts received limited coverage and were only occasionally used to scrutinise public health policy. Yet, our six-week online diary study with 175 participants identified a strong preference for expert views about how the pandemic was being handled. It showed audiences favoured a range of expert sources in routine reporting – balancing government appointed and independent experts – to provide evidence-based scrutiny of the executive’s decision-making. Overall, our findings contribute to a greater understanding of audience expectations, opinions, and experiences with broadcast news during a major public health crisis.

## Introduction

The outbreak of the COVID-19 pandemic produced a rapid rise of disinformation via social media and other informal networks of communication which largely questioned scientific knowledge and official guidance about the virus. Political leaders worldwide have also been held responsible for confusing, misleading, or even false claims about the virus, which were repeated in mainstream media (Hatcher, 2020). At the same time, biomedical experts such as virologists, epidemiologists, and public health scholars have played a key role in providing scientific advice to governments during the pandemic. In the UK, routine press conferences have dominated the news cycle with government’s scientific advisers having a visible and active role in the decision-making process. However, while UK government ministers emphasised on many occasions their reliance on a science-led approach, records suggest there were sometimes contrasting views between the government and its scientific advisers about the plans of action (Newton, 2020).

During a global health crisis, professional news media play a critical role in communicating clear and reliable information. This includes the editorial selection and representation of sources of knowledge and expertise which have the potential to either enhance or undermine public understanding of health policy and hold the government accountable for its decisions. Our study considers expert sources as a crucial feature of coverage during a major health crisis. Yet, while the editorial construction of experts has been widely assessed in coverage including in general health news (e.g. Stroobant et al., 2018) and pandemic reporting (e.g. Mellado et al., 2021), rarely has research combined a systematic content analysis of sourcing patterns with an assessment of public attitudes towards the sources speaking in the news. A vast body of research in recent years has been concerned with public perceptions of expertise (Dommett and Pearce, 2019) which have attracted renewed scholarly interest in the context of the coronavirus pandemic particularly in relation to the salient issue of public trust in expertise (Mihelj et al., 2022b). Our study draws on such a combination of perspectives to explore new empirical lines of inquiry about the relationship between audience expectations and news media practices. In doing so, ultimately, we aim to assess how well UK’s highly regulated broadcast media system serve the public during a public health crisis. We therefore conducted a systematic content analysis examining the distribution and range of sources used in the flagship UK evening television news programmes over a period of four weeks during April–May 2020. In doing so, we consider selection and construction of expert sources in UK television news coverage including the degree of scrutiny of government decision-making within the regulatory obligations to produce impartial journalism. At the same time, we examined audiences’ perceptions of media coverage of the pandemic through a six-week online diary study including questions concerning participants’ preferences for sources informing the coverage of the pandemic.

Our study contributes to broader debates in journalism studies concerning the media representation of expert sources by focusing on impartial media ecologies with public service regulation. It also contributes to a greater understanding of audience expectations, opinions and experiences with broadcast media content and its sources and has the potential to inform recommendations for enhancing the democratic and informative value of UK television journalism during a global health crisis.

### Experts and sourcing patterns in pandemic news

As a wide-ranging object of research in journalism scholarship, experts as news sources represent a diverse category of social actors perceived to possess neutral and factual knowledge which enables them to explain and interpret complex issues and events within the professional journalistic framework of objectivity (Albæk, 2011). When it comes to public health issues, the selection of credible sources of knowledge is of high importance for media organisations with obligations to deliver balanced, informative, and impartial information to their audiences.

The news reporting of infectious disease outbreaks has attracted significant scholarly interest over the years producing a vast body of research assessing different facets of coverage including sourcing patterns. Studies on the widely researched 2009 new H1N1 influenza virus (also known as “swine flu”) found a prevalence of the perspectives of public health authorities and biomedical experts in many countries’ media coverage (e.g. Hallin et al., 2020; Husemann and Fischer, 2015) which Briggs and Hallin (2016) attributed to a largely cooperative relationship between news media and health authorities. Yet, the authors also recognise that such consensual model of health reporting was not consistently registered across different epidemics or national contexts. Several studies have demonstrated how the mediatisation of pandemics is shaped by different factors including disease characteristics, media attention-cycles, journalism culture and political context (Mellado et al., 2021; Shih et al., 2011; Singer et al., 2020). For instance, Jung Oh et al. (2012) found that whilst the Korean press heavily relied on government sources, US newspapers reflected a wider range of perspectives with more emphasis on the government’s responsibility in resolving the 2009 H1N1 flu pandemic.

An emerging body of research on the news media coverage the coronavirus (COVID-19) pandemic across different national contexts and platforms has found that political actors have dominated the news reporting of the first phase of the pandemic (Hart et al., 2020; Hubner, 2021; Mellado et al., 2021). The prominence of politicians and government authorities as actors with decision-making power reflects in part the large scale of the COVID-19 pandemic and its wide-ranging impact across society. At the same time, many studies have drawn attention to the risks of a ‘politicization’ of the crisis particularly in polarised information environments where the mediatisation of the pandemic has reflected the partisan arguments of political elites at the expenses of an impartial reporting of the scientific advice (Apuke and Omar, 2021; Fox, 2021; Hart et al., 2020). This growing body of research on the ongoing health crisis, while building on the scholarly work of past disease outbreaks, highlights the importance of assessing sourcing patterns within a contextualised understanding of information environments and journalism cultures. However, while a significant body of recent studies have concentrated on polarised media systems with a strong focus on the US media and political context, comparatively limited research has examined impartial news environments with a public service ethos.

In general, despite acknowledging the crucial role of mainstream media in the reporting of public health (Briggs and Hallin, 2016), broadcast news media remain a sparse area of empirical investigation particularly with regards to studies of sources which tend to concentrate on print and online news outlets. Yet, studies that have assessed broadcast content seem to point to a more equal distribution of politicians and scientists in television news than in the press (Hart et al., 2020). This reinforces the need for further research assessing the type and range of sources in broadcast news environments with a focus on the editorial construction of experts and audience perceptions of such conventions during a major health crisis.

### Public trust, audiences, and government accountability

During a pandemic, public trust in experts and politicians is crucial for effective policymaking (Cairney and Wellstead, 2020). Despite experts and scientists being the target of anti-elitist sentiments associated with a rise in populism in many countries in recent years (Mede and Schäfer, 2020), survey data found broadly positive public attitudes towards experts across many countries (Dommett and Pearce, 2019) persisting throughout the pandemic (Nielsen et al., 2021). Yet, the highly politicised nature of the crisis has raised public concerns about the independence of the advisory process in some countries (Mihelj et al., 2022b). At the same time, while the statement ‘following the science’ has been used by UK politicians on many occasions, research has revealed discrepancies between government and scientific advisers in understanding of the science (Colman et al., 2021) or in the choice of course of action (Newton, 2020). Reported public confusion with government’s COVID-19 measures such as lockdown rules across the UK nations (Dodd and Pidd, 2020) further highlights the crucial role of news media in delivering informative, accurate and impartial coverage about government decision-making. In this, experts possess knowledge that is often highly relevant for interpreting public policy issues (Albæk, 2011: 346) through the delivery of evidence-based assessments of government’s decision-making. Yet, while a body of scholarship has long reflected on the complex relationship between experts and policymakers both at normative (Pielke, 2007) and empirical level (Coleman et al., 2021), rarely has research examined the editorial role of expert commentary on government’s policy. Furthermore, despite several studies have provided quantitative assessments of sourcing patterns in pandemic coverage (Mellado et al., 2021; Singer et al., 2020), seldom are they assessed against audience reception of voices speaking in the news.

Existing research on media audiences during the coronavirus pandemic has heavily relied on quantitative survey studies with particular interest in news consumption patterns. Findings from global surveys showed an increase in TV news consumption (Nielsen et al., 2021) which Van Aelst et al.’s (2021) study associates to pre-existing levels of trust in legacy media. Quantitative surveys have also been used to assess the influence of partisanship on public attitudes to health policies (Gadarian et al., 2021). Studies drawing on qualitative methodologies have been comparatively more sporadic, yet they have contributed to nuanced understandings of news habits in lockdown (Mihelj et al., 2022a) and of public perception of trustworthiness. For example, in a study drawing on diaries and interviews, Mihelj et al., 2022b found that lack of independence from political elites was a key factor contributing to distrust in experts in relatively new Eastern European democracies. Further studies have demonstrated that television audiences display sophisticated critical readings of media coverage(Kyriakidou et al., 2022). In their study of Spanish public television, Villena-Alarcón and Caballero-Galeote (2020) found that audiences felt coronavirus media coverage lacked impartiality and would welcome less politicised content. Similary, Apuke and Omar’s (2021) examination of Nigerian television news illustrates that audiences displayed strong awareness of political economy factors such as state ownership shaping media coverage and were critical of the partisan divide observed across private and government TV stations at the expenses of a neutral and factual reporting of the severity of the virus.

While studies focusing on broadcast news remain sparse, existing research during the COVID-19 pandemic highlights the importance of understanding the needs of audiences based on their critical readings of coverage within contextualised assessments of media environments.

### The UK case study and research questions

The UK’s public service media system and political culture, in our view, represents an important case study to explore sourcing patterns in an impartial news environment and less partisan culture than many other countries. At the same time, as research has shown that during a pandemic a public service ethos does not automatically translate into news output that routinely and most effectively hold the powerful to account (Cushion et al., 2021) and best serve the information needs of audiences (Villena-Alarcón and Caballero-Galeote, 2020), our study has the potential to enhance the legitimacy of public service journalism in times of crisis through recommended editorial practices. As previously discussed, experts have long been an object of research which has attracted renewed attention in the context of the COVID-19 health crisis. Yet, systematic assessments of sourcing patterns in news media do not always contextualise the quantitative findings, such as the extent to which experts scrutinise government decision-making in news reporting. Our study develops a rigorous understanding of the role of expertise in coverage and the public’s response to it. Specifically, we examine UK television news coverage at the height of the first wave of the COVID-19 pandemic by combining a systematic content analysis of sources with a six-week diary study exploring and assessing audiences’ perceptions about source selection in UK television coverage with particular attention to audience views about experts in the news. In doing so, our study will reveal how audiences respond to media coverage and explore how the UK’s overarching public service broadcast ecology serves the information needs and sourcing preferences of audiences. We will also explore the editorial selection of sources across UK broadcasters with different regulatory frameworks and funding models.

More specifically, the study aims to explore the following research questions:

RQ1: What is the type, range and frequency of different sources used in UK television coverage of COVID-19 at the height of the first wave of the pandemic?
RQ2: How are ‘experts’ represented in relation to distinct professional roles, including their positioning within the bulletin, the type of story they feature in and degree of scrutiny they provide of the government’s handling of the crisis?
RQ3: How do news audiences perceive the type and role of sources informing television bulletins during the pandemic? Do their views change over the six-week study?

## Methodology

### Content analysis

The content analysis systematically examined television news output over a period of four weeks (14 April to 10 May 2020 excluding Easter Monday). The sample included the five main UK evening bulletins: BBC News at Ten, ITV News at Ten, Channel 4 at 7pm, Channel 5 at 5pm and Sky News at Ten. While all UK broadcasters are subject to the same legal requirements to be accurate and impartial, they have different Ofcom-regulated license obligations in the provision of news: while the BBC is the main public service broadcaster, ITV, Channel 4 and Channel 5 are commercial public service broadcasters with contrasting license agreements about their news provision. In contrast, Sky News is a commercial broadcaster with no public service obligations.

To establish a fair comparative assessment of the news programmes we only coded the first 25 minutes for Channel 4 bulletins (excluding headlines) so that it was approximately the same length as other programmes. It should also be noted that the weekend editions varied in length with Channel 5 and ITV shorter than the other broadcasters (5 and 20 minutes respectively) compared to the other bulletins (typically 20–25 min). The unit of analysis refers to every pandemic-related television item over the sample period. This was based on a news convention rather than story (e.g. pre-edited package with reporter on location). The content analysis study generated a total of 1347 items across five broadcasters with 1259 items that focused on the coronavirus pandemic. Table 1 shows the spread of the sample including slight imbalance in the share of items across the period under analysis with more items on BBC and Sky News. This was largely due to the length of items being longer on Channel 4 and overall airtime on ITV and Channel 5 being shorter than other broadcasters during weekends.

**Table 1.** The percentage of news items in the sample across different UK news bulletins.

Programme	COVID-19-focused items (N)
BBC at 10pm	93.9% (307)
ITV at 10pm	95.7% (247)
Channel 4 at 7pm	97.4% (190)
Channel 5 at 5pm	94.9% (222)
Sky News at 10pm	88.0% (293)
Total	93.5% (1259)

Two researchers coded all news items on the UK’s flagship evening bulletins. The content analysis was wide-ranging, but for the purpose of this article we focused on the variables concerning the focus of this study. We identified 2300 sources speaking directly in the 1259 news items about the pandemic. Alongside frequency proportions for type of sources across the news programmes, a more detailed analysis of all expert sources was carried out to examine the editorial construction of expert voices in the news through an assessment of the broadcasters’ framing of expert sources including the positioning within the item and the subject category of the story. Furthermore, to assess the degree of scrutiny of the government’s response to the pandemic, we coded whether and to what extent – either implicitly or explicitly – the different types of expert sources included some degree of scrutiny. After re-coding approximately 10% of the entire sample, the variables achieved a high level of inter-coder reliability according to Cohen’s Kappa (see Appendix).

### Diary study

The diary study complemented the content analysis by exploring how news audiences were responding to the news coverage at the height of the first wave of the pandemic over the course of six weeks (16 April–27 May 2020). While diary studies have a long tradition in the social sciences and in psychology as a methodological tool for audience research (Bolger et al., 2003), have only recently started being adopted within journalism studies (Cushion et al., 2021; Mihelj et al., 2022a). We selected a diary study enabling both qualitative and quantitative assessment of audience’ news media consumption habits and experience with news output for two main reasons. Firstly, it enabled us to assess people’s knowledge, understanding and engagement with the health crisis at key moments of the pandemic in its early phase as well as to explore any changes over the 6-week period. The study started during a period of extended lockdown and terminated with the gradual easing of restrictions across the UK. Therefore, the self-reflective nature of the diary study allowed us to assess potentially shifting perceptions across a period of rapidly changing policy guidance. Secondly, a remote digital diary study provided a valid alternative to in-person audience research during a time of strict social distancing measures.

We recruited an initial mix of 200 people in the UK via an online recruitment company, Prolific. Our diary sample was not fully representative of the UK population as it included a larger number of female participants than male (146 vs. 54). However, it provided a qualitative assessment of a wide range of views and opinions. Over six weeks, respondents were asked to complete two diary entries a week (12 in total) on a wide range of issues, ranging from their media consumption habits and their reflections on the informative value of TV news media coverage, to their knowledge and understanding of government lockdown measures. Our sample represented a demographic mix of news audiences who relied to a large extent on television news bulletins during the health crisis. Media consumption habits of our participants were assessed regularly throughout the study, and we found that the BBC was the most watched and trusted broadcaster with half of respondents watching it every day or most days in the last week. This reflected larger, representative surveys published around the same time (Ofcom, 2020).

For this study, we focus on entry 5 (30 April–3 May 2020) and entry 11 (21–24 May 2020), where respondents were asked to share their views on the range of sources represented in TV news reporting of the pandemic. Attrition was experienced over the six weeks resulting in 175 valid responses in entry 5 and 161 in entry 11.

The question we asked in both entries was:

“Thinking about TV news bulletins, are there people you would like to see more of and hear more from? Please share who and why."

In entry 11 we also invited respondents to reflect more generally on whether their views of people they would like to hear more in news coverage had changed over the period of the diary study. To analyse the diary responses, we combined quantitative and qualitative analysis. Source categories of preference were quantitatively coded using a combination of automatic text search via NVivo and manual coding to confirm semantic validity. We then adopted a qualitative approach to identify and code wider themes emerging from the participant responses according to the principles of applied thematic analysis (Guest et al., 2012). Themes – which included ‘trust’ and ‘political independence’ – were developed inductively after several readings of the data and by paying close attention to participants’ perceptions of sources.

## Findings

### An overview of sources in COVID-19 television news coverage

Table 2 shows the top 10 sources speaking directly in the news items accounting for the 81.7% of the total sources in our sample (*N* = 2300). However, we found significant variations in the proportion of sources across each broadcaster: the BBC featured the most sources (*N* = 660) followed by Sky News, while Channel 5 used sources least frequently (*N* = 298). This variation reflects the different use of conventions between broadcasters: Channel 5 had by far the highest number of standalone anchor-only pieces (47%) which typically do not use any source – as well of the different lengths of the bulletins’ weekend editions. This perhaps reflects Channel 5’s far more limited budget than other broadcasters, relying on the anchor – rather than a range of specialist journalists – to communicate coverage.

**Table 2.** Frequency and type of top 10 sources in television news coverage of the pandemic (*N* = 2300).

Source category	BBC	ITV	Channel 4	Channel 5	Sky News	Total
Politicians	16.7% (110)	21.6% (94)	27.4% (101)	15.8% (47)	22.3% (120)	20.5% (472)
Citizens	16.4% (108)	11.9% (52)	13.8% (51)	14.8% (44)	14.2% (76)	14.4% (331)
Healthcare workers	11.1% (73)	8.7% (38)	11.4% (42)	13.8% (41)	5.6% (30)	9.7% (224)
Experts	8.6% (57)	8.3% (36)	12.7% (47)	5.4% (16)	11.7% (63)	9.5% (219)
Business	7.9% (52)	7.3% (32)	6.2% (23)	7.7% (23)	7.1% (38)	7.3% (168)
Patients and relatives	9.2% (61)	12.2% (53)	4.9% (18)	2.3% (7)	5% (27)	7.2% (166)
Charity/NGO	3.6% (24)	2.1% (9)	1.9% (7)	4.4% (13)	3.5% (19)	3.1% (72)
Trade associations	2.1% (14)	3% (13)	3.8% (14)	5.4% (16)	2% (11)	3% (68)
Care homes	5.8% (38)	5% (22)	5.7% (21)	4% (12)	4.1% (22)	5% (115)
Volunteers/fundraisers	1.2% (8)	1.4% (6)	0.3% (1)	4.7% (14)	2.8% (15)	1.9% (44)
Other	17.4% (115)	18.6% (81)	11.9% (44)	21.8% (65)	21.6% (116)	18.3% (421)
Total	100% (660)	100% (436)	100% (369)	100% (298)	100% (537)	100% (2300)

Politicians were the largest source category across all broadcasters, regularly featuring in the opening news items of the bulletins as prominent actors typically dominating and defining the news agenda. When political sources are broken down, Conservative politicians – representatives of the incumbent UK government – were the most featured source accounting for more than half (54.9%) of all political sources. In contrast, the voices of the main opposition party (Labour) received limited airtime (17.8%) which was only just more appearance than that of international politicians (17.6%). The voices of ordinary citizens reacting to government’s measures represented the second largest category and were a routine part of news coverage reflecting television news values of emotion and identification (Verhoeven, 2008). The testimonies of healthcare professionals often from hospital or even ICU settings acting as witnesses on the reality of the virus were the third largest source category. Therefore, we found that political sources, citizens and healthcare professionals outweighed the category of ‘experts’ which accounted for only 9.5% (*N* = 219) of our analysis of 2300 source.

When looking at the breakdown of specific type of expert sources (see Table 3), we found that the majority of experts (*N* = 138, 63%) speaking in the news were health and biomedical experts such as virologists, epidemiologists, public health scholars, biomedical researchers affiliated to scholarly or research institutions but with no advisory role in government. As such we refer to these sources as ‘independent’ scientific experts. Members of the Scientific Advisory Group for Emergencies (SAGE) – the UK government’s body that provides scientific and technical advice to support government’s decisions during the pandemic – made up 26.9% (*N* = 59) of the total experts in our sample. A further and much smaller ‘expert’ subcategory included academics from non-medical disciplines such as social scientists with contributions about the wider societal impact of the pandemic. Sky News and the BBC were the two broadcasters that more frequently featured the direct contributions of experts in news reports followed by Channel 4, ITV, and Channel 5.

**Table 3.** Breakdown of expert sources in television news coverage of the pandemic (*N* = 219).

Expert source	BBC	ITV	Channel 4	Channel 5	Sky News	Total
Independent scientist/health researcher	70.2% (40)	58.3% (21)	61.7% (29)	75.0% (12)	57.1% (36)	63.0% (138)
Government’s scientific adviser (SAGE)	22.8% (13)	30.6% (11)	25.5% (12)	12.5% (2)	33.3% (21)	26.9% (59)
Non-medical academic	7.0% (4)	11.1% (4)	12.8% (6)	12.5% (2)	9.5% (6)	10.0% (22)
Total	100% (57)	100% (36)	100% (47)	100% (16)	100% (63)	100% (219)

Beyond individual scientific experts, we found a limited range of other sources of authoritative knowledge and expertise. Intergovernmental organisations accounted for just 0.8% of total sources with the World Health Organization (WHO) only featuring 10 times in our sample. This is surprising given the key role the UN agency played in issuing COVID-19 guidance at global level and significantly contrasts with existing literature about its prominence in the H1N1 pandemic (cf. Husemann and Fischer, 2015). Similarly, economists (0.3%) and think tanks (0.3%) barely registered in coverage despite the severe economic and societal impact of the pandemic.

### Selection and construction of expert voices

While independent experts featured more frequently than government scientific advisers, members of the Scientific Advisory Group for Emergencies (SAGE) tended to have a more prominent, ‘primary-definer’ position in the bulletins. For example, they often featured speaking alongside government cabinet ministers at daily press briefings, explaining policy decisions in relation to the scientific evidence informing policy measures, and the way the virus was spreading across the UK. Examining the frequency of type of expert sources per story topic, we found that SAGE members featured in items about the UK government’s response to the crisis including general UK government-focused updates on the latest or forthcoming announcements (20.3%), decisions taken to extend or ease lockdown restrictions (22.0%), and National Health Service (NHS) and healthcare generic issues (17.0%). Only 10.2% of SAGE sources featured in items about COVID-19 research development stories. By contrast, the voices of independent scientists extensively informed science-focused reporting on research development in vaccine, track and tracing, treatment research (45.6%) or stories focusing on NHS/healthcare and testing (20.3%) and lockdown measures (22.0%). Interestingly, only 5.8% of the independent scientific sources appeared in stories specifically focusing on the government’s response to the pandemic. This meant there were limited opportunities for independent experts to provide a commentary on the government’s handling of the crisis.

#### Expert scrutiny of government’s decisions

To further understand to what extent expert sources scrutinised the government’s agenda, we assessed the degree of their questioning of policy. In our subsample of items involving 219 expert sources, we found only 61 instances (28%) where experts were used to scrutinise the government’s decision-making. Most instances of scrutiny (80%, *N* = 49) were advanced by independent experts and largely concerned testing capacity, the government’s handling of the pandemic in general terms and contact tracing programme.

As Table 4 shows, there are variances across broadcasters: Channel 4 featured half of the experts’ questioning of government’s policy (*N* = 31) and was the only broadcaster that featured independent scientists explicitly scrutinising the government’s management of the crisis.

**Table 4.** Proportion of news items with explicit or partial/implicit questioning of UK Government decisions by type of expert sources (*N* = 61).

Degree of scrutiny per programme	Independent scientist/health researcher	Government’s scientific adviser (SAGE)	Non-medical academic	Total
Channel 4	**55.0% (22)**	**27.3% (3)**	**60.0% (6)**	**50.8% (31)**
Explicit	12.5% (5)	9.1% (1)	—	9.8% (6)
Partial/Implicit	42.5% (17)	18.2% (2)	60.0% (6)	41.0% (25)
ITV	**15.0% (6)**	**27.3% (3)**	**10.0% (1)**	**16.4% (10)**
Explicit	5.0% (2)	18.2% (2)	—	6.6% (4)
Partial/Implicit	10.0% (4)	9.1% (1)	10.0% (1)	9.8% (6)
Sky News	**10.0% (4)**	**36.4% (4)**	**10.0% (1)**	**14.7% (9)**
Explicit	2.5% (1)	—	—	1.6% (1)
Partial/Implicit	7.5% (3)	36.4% (4)	10.0% (1)	13.1% (8)
BBC	**12.5% (5)**	**9.1% (1)**	**10.0% (1)**	**11.5% (7)**
Explicit	—	9.1% (1)	—	1.6% (1)
Partial/Implicit	12.5% (5)	—	10.0% (1)	9.8% (6)
Channel 5	**7.5% (3)**	—	**10.0% (1)**	**6.6% (4)**
Explicit	2.5% (1)	—	—	1.6% (1)
Partial/Implicit	5.0% (2)	—	10.0% (1)	4.9% (3)
Total	**100% (40)**	**100% (11)**	**100% (10)**	**100% (61)**

For example, on the 19^th^ of April, Sir David King – Emeritus Professor in Physical Chemistry at the University of Cambridge was asked by a Channel 4 reporter to elaborate on his statement that Britain was not prepared to respond quickly to the pandemic. In his *explicit* critical assessment of the government’s handling of the crisis, King identified two main factors. Firstly, austerity measures that the Conservative government introduced in 2010 which “*meant that all the preparations to risk-manage for the feature were effectively jettisoned in favour of dealing with current crises because there simply wasn’t the money available”.* And secondly, “… *the command-and-control policy which also changed in 2010 where Number 10 and its communication people control who says what in the public domain* (...) and calling for a clearer and transparent communication of the scientific advice (Channel 4, 19^th^ April).

Among expert sources, most instances of scrutiny were voiced by independent experts (N 50) with no current role in government, while just 11 featured SAGE members questioning the government’s response. On the 5^th^ of May, for example, after seven weeks of lockdown, the UK recorded Europe’s highest number of deaths and all broadcasters – apart from Channel 5 – included an interview clip featuring Government Chief Scientific Adviser Sir Patrick Vallance in an informal setting raising the issue of testing capacity and *implicitly* admitting that the government had failed to ramp up coronavirus testing quickly enough in the early stages of the pandemic:

In the early phases, and I’ve said this before, I think if we’d managed to ramp testing capacity quicker, it would have been beneficial, and... You know, for all sorts of reasons, that didn’t happen, and I think it’s clear you need lots of testing for this.

(BBC News at Ten, 5^th^ May)

On the same day, Sky News, as part of a reporter package, featured a clip with Professor Jenny Harries – Deputy Chief Medical Officer for England and SAGE member – being questioned by Chair of the Health and Social Care Select Committee, Jeremy Hunt, on whether she thought “*it was not appropriate to test in the community*”. Professor Harries responded by *implicitly* casting doubt over the government/SAGE’s approach at the time: “*If we had unlimited capacity and ongoing support beyond that, then perhaps, we would choose a slightly different approach”*.

Overall, the analysis showed that the government’s scientific advisers were not routinely used to scrutinise the government’s advice and, when they were, the claims included in the reports tended to be implicit instances of questioning. While independent experts more frequently and explicitly questioned the government’s response to the pandemic, our findings reveal an imbalance across broadcasters with Channel 4 devoting far more attention to the explicitly critical voices of experts than any other broadcaster. Finally, when analysing the positioning of expert sources within bulletins, we discovered that while SAGE members had a ‘primary definer’ role within items focusing on the government’s decisions, the voices of independent experts tend to be limited to items about scientific research development with few opportunities for direct scrutiny of policy decisions with the exception of a narrow range of visible eminent public health scholars.

### Which sources would television audiences like to hear more from?

**Figure 1.**
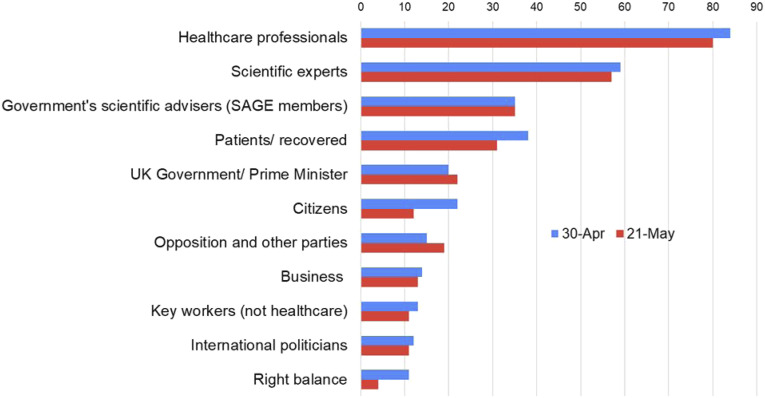
Visualisation of top 10 source categories mentioned by participants in their responses as preferred news sources per date question asked. 30 April: *N* = 346 source categories mentioned; 21 May: *N* = 326 source categories mentioned.

To explore the role of television news in people’s understanding of the health crisis and the significance of news sources, we explored audience attitudes towards them at a key point in the pandemic. At two different moments in time (30 April–3 May 2020 and 21–24 May 2020), our diary participants were asked to share which sources they would like to hear more from in television news. Most of the responses recognised the informative value of a range of voices contributing to the coverage and named specific social actors that they thought deserved more airtime. As Figure 1 shows, for most categories there was a striking consistency across the two dates.

By examining participant responses, we identified differences between who broadcasters selected to inform coverage about the pandemic compared to who the public wanted to hear from. Whilst politicians were the dominant source across all broadcasters, we found that our participants most valued the voices and insights of healthcare professionals. As sources with “*first-hand experience in dealing with the virus*” as one participant put it, frontline nurses and doctors were viewed as social actors with practice-based knowledge derived from witnessing the effects of the pandemic on patients. In their responses, participants pointed out that healthcare staff’s first-hand accounts can serve as a warning to the seriousness of the disease and act as a deterrent to the breaching of restrictions.

Experts – largely scientists – were the second most preferred source for our respondents. The factual and evidence-based knowledge of expert sources was regarded as an important quality that would help the respondents navigate through the blizzard of dubious information that quickly emerged during the pandemic:

I would like to hear more from scientific advisors and scientists. I think that they will be the most forthcoming in providing the facts of the situation. I think that they will also be able to provide a rational view of the current situation and will back up their opinions with scientific facts.

Despite the question we asked did not specifically mention ‘trust’ – as we kept the wording purposefully broad – the quality of ‘trustworthiness’ was frequently brought up by our participants:

I would definitely like to hear more from scientists, professionals in the field, doctors and WHO. Because I trust them the most and believe they have the real insight and knowledge into what is going on and how to tackle it.

The perceived trustworthiness and impartiality of expert advice was juxtaposed by some participants with the partisanship of "*political arguments"* as one participant put it. These responses often expressed distrust towards government’s communication of public health guidance, which was perceived as being driven by a political agenda rather than by the science:

I would like to hear more from scientists because then politics does not get in the way of the real facts. I trust scientists the most out of everyone as they have no reason to share false information and the public health is in their best interest.

Members of the UK Scientific Advisory Group of Emergencies (SAGE) were also mentioned as a category of experts that our respondents would like to hear more from. This was largely because they wanted to better understand the scientific evidence behind policy decisions. As one participant put it: “*We hear about what decisions have been made but not the reasons behind those decisions”*. A few participants pointed out that they would welcome a wider range of scientific experts to hold government claims and decisions to account:

Hearing from members of SAGE may be beneficial as well as other medical and scientific advisors. As the government claims to be following the science, other scientists should also be able to evaluate the evidence, not so as to undermine actions, but so as to see if the actions the government take are in line with scientific advice.

However, the issue of independence of scientific advisers was also raised in relation to expressing a preference for expert voices free from political interference:

I would like to hear more from scientific advisers independent of the government for neutral, science-led information on the virus and the effectiveness of measures put in place.

Although members of the government did not feature high among the sources our research participants valued, a few expressed an interest in hearing more from them as well as the Prime Minister Boris Johnson himself given his absence due to recovering from the virus.

A small but significant number of our participants pointed out that the voices of the opposition and other parties were not sufficiently covered in the news bulletins. Indeed, we found a slightly increased interest in a wider range of political sources when we asked respondents for a second time about their preferences for who they would like to hear from. In this latter diary entry, fewer participants thought that television news coverage represented the ‘right balance’ of source types as Figure 1 shows. To further explore this, we explicitly asked our participants whether they thought their views about who they would like to hear more of in television news had changed over the course of six weeks. Whilst 98 participants responded their views hadn’t changed and 13 did not provide a valid answer, 50 respondents disclosed that they now had a different opinion of who they would like to hear more of compared to their early responses. A few of these mentioned – as a contributing factor – the way the easing of the lockdown was being dealt with by the government. The inconsistent messages from government had left them confused and interested in hearing more from sources that can supply further background information or scrutiny of government’s decisions:

Yes, it has [changed], given that I have been hearing a lot of criticism with regards to how the government is handling the situation. As such, I would like to know whether there have been critical mistakes made and this I would like to be supported by evidence which I can trust to be reliable.

Sentiments of a declining trust in the UK government throughout the early months of the pandemic are consistent with findings looking into audience attitudes at the same point in time (Fletcher et al., 2020). In contrast, sources of expertise including healthcare staff as well as scientific experts remained consistently valued by participants across the six-week study. Furthermore, we found many respondents had a preference for independent and impartial experts in television news because they were seen as being able to effectively scrutinise policy decisions and hold the government to account.

## Rethinking source practices: towards more expert perspectives

The study examined the role of experts in UK television news at the start of the coronavirus pandemic by analysing both how they were used in coverage and perceived by news audiences. Our systematic content analysis discovered that experts accounted for just 9.5% of all sources over the four-week period while political actors dominated coverage across broadcasters. This reflects research findings across different national contexts pointing to a trend of politicised news coverage of the COVID-19 pandemic (e.g., Hart et al., 2020; Mellado et al., 2021) contrasting with the prominence of the biomedical perspective registered in the news reporting of past disease outbreaks (Hallin et al., 2020). While research on the mediatisation of the coronavirus pandemic has largely concentrated on polarised media environments, our study found that the UK’s public service media ecology with obligations to deliver accurate, factual and impartial reporting is not immune to the prevalence of political process at the expense of scientific commentary.

When government’s scientific advisers and other experts did feature, our study found that UK broadcasters provided limited opportunity for expert commentary on the government’s response to the pandemic. Our analysis also revealed differences across broadcasters. While BBC and Sky News featured most contributions of experts, Channel 4 presented a wider range of expert sources and more frequent and explicit expert scrutiny of the UK government’s handling of the pandemic. This suggests that besides differences in resources and the structural format of bulletins enabling longer interviews to be aired, there were distinct editorial strategies concerning government’s accountability which informed the selection of experts and interview questions. Overall, our detailed analysis of the editorial construction of experts pointed to a reluctance to routinely draw on independent experts to scrutinise government decision-making. Yet, our diary study showed it was expertise that our participants valued and trusted most reflecting broadly healthy levels of trust in expert sources as registered in larger studies (Nielsen et al., 2021)*.* Furthermore, as the most valued and trusted source, healthcare professionals, represented a practice-based form of expertise that participants perceived as reliable and free from political interference. Although diary respondents recognised the need for government perspectives to feature in pandemic coverage, political sources were often perceived as partisan and therefore less credible. Instead, many favoured greater scrutiny of political decisions by scientific experts – including those with advisory roles in government – in order to better understand how the pandemic was being handled and the science behind government decisions.

Our UK case study makes an important intervention into scholarly debates about the distribution and role of sources in news coverage with particular focus on the editorial representation of expertise. In this, it highlights the importance of a balanced range of non-partisan knowledge sources to improve public response to a major health crisis (Flores et al., 2022). Ultimately, our findings illustrate a discrepancy between audience expectations and journalistic practices. Our participants would welcome more specialist knowledge than currently exists in news coverage and our findings call for a balanced range of independent experts and voices from the government’s advisory board to interpret and scrutinise the executive’s decisions. We argue that in order to fulfil their informational role during a public health crisis, broadcasters with public service obligations need to rely more extensively on independent experts to better serve their wide audiences.

As far as limitations are concerned, our study is limited by the constraints of the sample period which precludes a more longitudinal assessment of media coverage and of public perceptions of social actors at different times in the pandemic. Furthermore, future studies could incorporate further platforms within the UK public service remit or establish a cross-national comparison of television news and their audiences in relation to sourcing patterns. Nevertheless, this study offered a valuable insight into the editorial choices of UK broadcasters with the potential to inform recommendations that enhance the legitimacy of public service media by responding to audience needs. Its findings contribute to a greater understanding of audience expectations, opinions, and experiences with broadcast media content during a public health crisis. We would recommend future studies combine both content analyses and audience perspectives about source preferences in order to identify how journalism can enhance public knowledge and understanding of crucial events, issues and policy.

## Appendix

**Table:**

Variable name	Level of agreement, Cohen’s kappa (CK) in brackets
Convention type	99.3% (0.99 CK)
COVID-19 focus (yes/no)	100%
Political sources	99.6% (0.98 CK)
Vox pop sources	99.2% (0.98 CK)
Other/expert sources	98.3% (0.97 CK)
Author of criticism	95% (0.92 CK)
Object of criticism	94.1% (0.93 CK)
Extent of criticism (implicit/explicit)	94.1% (0.88 CK)
